# Supplementary material for: Evolutionary modelling indicates that mosquito metabolism shapes the life-history strategies of Plasmodium parasites
Source: Nat Commun. 2023 Dec 14;14:8139. doi: 10.1038/s41467-023-43810-1 (PMC10721866; doi:10.1038/s41467-023-43810-1)
Supplement: Supplementary file 1 — Supplementary Information [file 41467_2023_43810_MOESM1_ESM.pdf]

Evolutionary modelling indicates that mosquito metabolism shapes life-history strategies of *Plasmodium* parasites

Paola Carrillo-Bustamante[1,*], Giulia Costa[1], Lena Lampe[1,2], and Elena A. Levashina[1,*]

[1]Vector Biology Unit, Max Planck Institute for Infection Biology, 10117 Berlin
[2]current address: Physiology and Metabolism Laboratory, The Francis Crick Institute, NW11AT London
[*]carrillo@mpiib-berlin.mpg.de, levashina@mpiib-berlin.mpg.de

SUPPLEMENTARY INFORMATION

SUPPLEMENTARY FIGURES

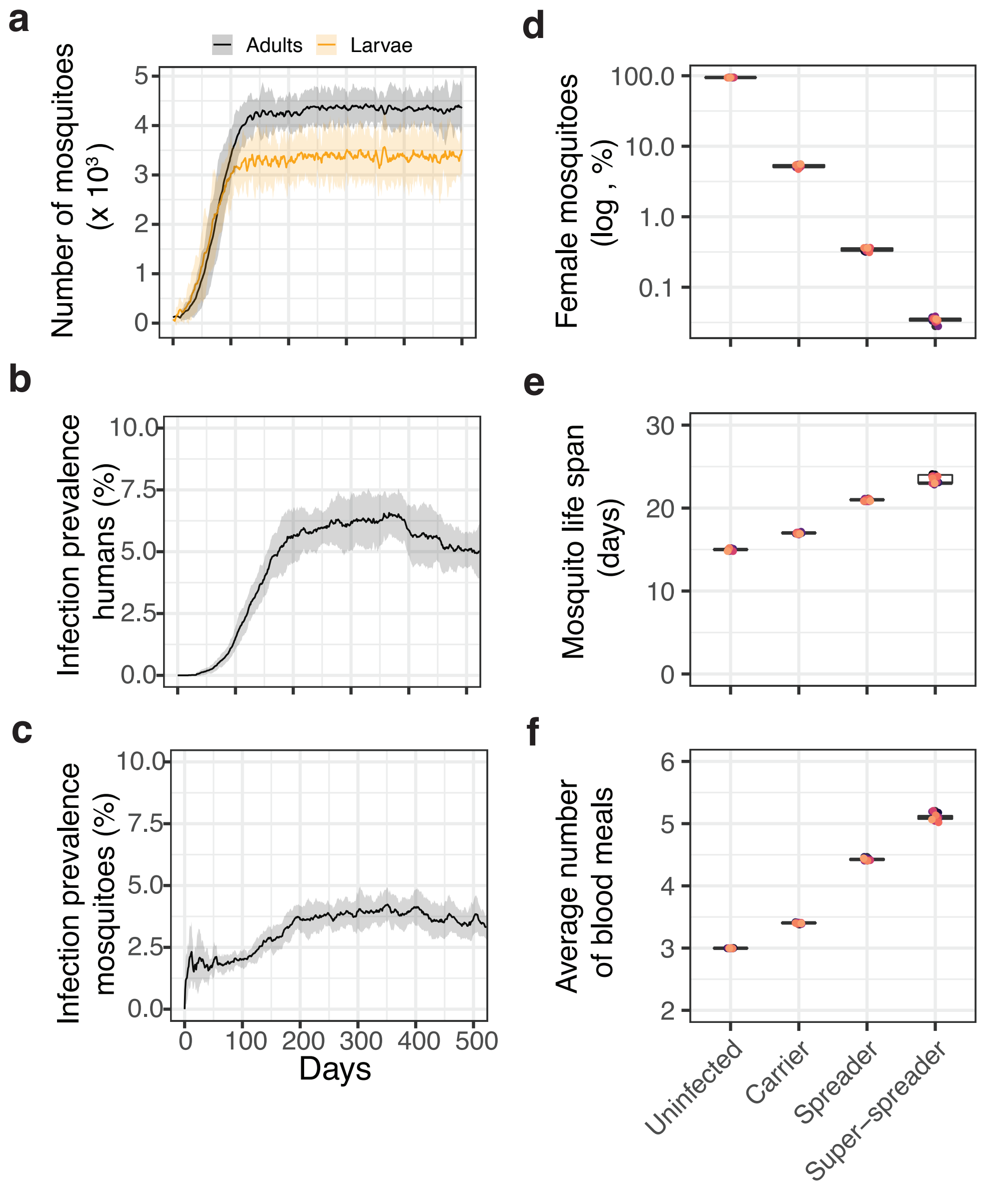

Supplementary Figure 1. Events of malaria transmission are rare and caused by long-lived mosquitoes. Simulations of 15 mosquito populations that grow with the start of a rainy season in the vicinity of human hosts. **a** Initialized with 100 larvae and 100 adults, the mosquito populations readily grow over time, reaching a steady state defined by their carrying capacity. Infection prevalence given by the number of *new* infections in humans (**b**) and vectors (**c**). At the beginning of every simulation, 20% of humans are assumed to be asymptomatic carriers. With the growing mosquito population, parasite transmission increases, infecting on average more than 6% of humans, and 4% of vectors. Solid lines depict the average of N= 15 simulations, and the shaded areas show the standard deviation. **d** Percentage of mosquitoes in every category of infection status: uninfected (94.41%), carriers (i.e. mosquitoes that do not transmit the parasite, 5.21%), spreaders (mosquitoes that infect one human host, 0.35%), and super-spreaders (mosquitoes that infect more than human host, 0.03%). The percentages were calculated by counting the number of mosquitoes throughout the entire simulation. **e** Life span of mosquitoes calculated in every infection category by measuring the median life span per simulation. **f** Mean number of blood meals taken per mosquito. Box plots show the median with first and third quartile, whiskers depict min and max values. Colors represent the replicates of each independent simulation.

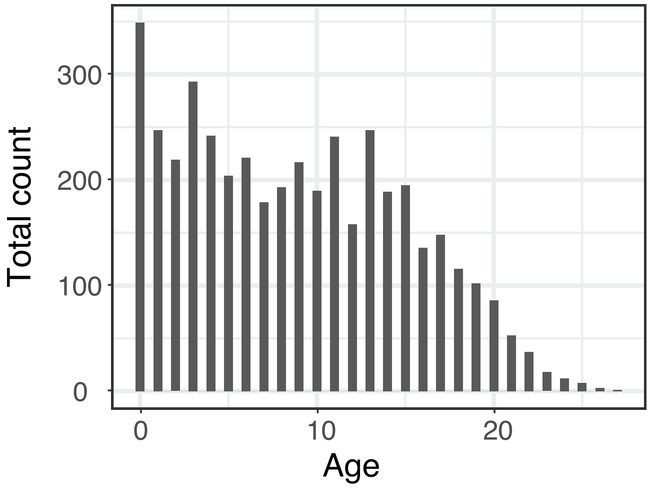

Supplementary Figure 2. Mosquito age-distribution in the individual-based model. We model the mosquito death event as an age-dependent process described with a Gompertz distribution with shape $x = 0.16$ and scale $b = 0.00761$. Although this death rate function was fitted to mosquitoes reared under laboratory conditions, the emerging age-distribution is remarkably similar to mosquitoes sampled in the field[1]. Representative example of one (out of N=15) simulated mosquito population after reaching steady-state,. i.e., after 300 days.

Supplementary References

1. Siria, D. J. *et al.* Rapid age-grading and species identification of natural mosquitoes for malaria surveillance. *Nat. Commun.* **13**, 1501, DOI: 10.1038/s41467-022-28980-8 (2022).

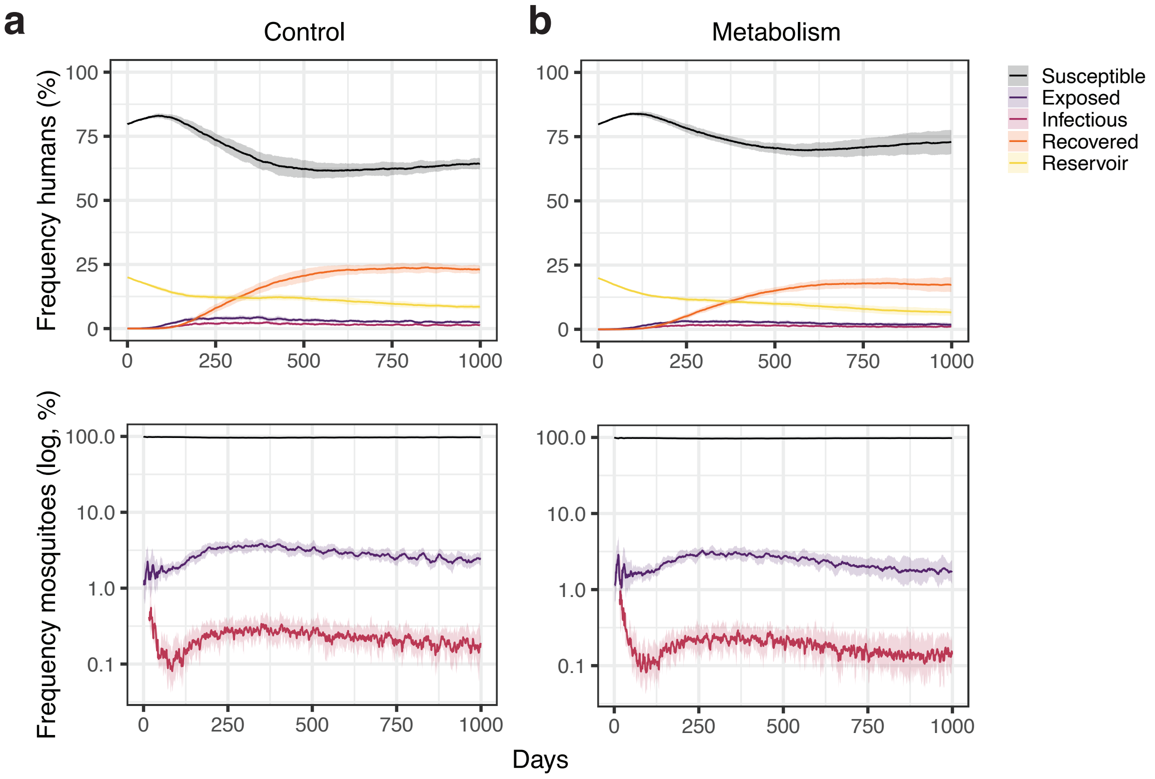

Supplementary Figure 3. Infection dynamics of humans and mosquitoes. Proportion of susceptible (black), exposed (purple), infectious (pink), recovered (orange), and initial reservoir (yellow) humans (top rows), and susceptible, exposed, and infectious mosquitoes (bottom rows). The chosen parameters result in stable infection dynamics. Importantly, including mosquito metabolism (right column) does not cause any substantial change in these infection dynamics. Solid lines depict the average of N= 15 simulations, and the shaded areas show the standard deviation.
